# Cerebellar pilocytic astrocytoma: predictors of recurrence based on MRI morphology—a single-centre experience

**DOI:** 10.1007/s00381-024-06733-w

**Published:** 2024-12-28

**Authors:** Katherina Grin, Amedeo Azizi, Christine Haberler, Andreas Peyrl, Gregor Kasprian, Thomas Czech, Karl Rössler, Johannes Gojo, Christian Dorfer

**Affiliations:** 1 Department of Neurosurgery, Medical University of Vienna, Währinger Gürtel 18-20, 1090 Vienna, Austria; 2 Department of Pediatrics and Adolescent Medicine, Medical University of Vienna, Vienna, Austria; 3 Department of Biomedical Imaging and Image-Guided Therapy, Medical University of Vienna, Vienna, Austria; 4 Department of Neurology and Neurochemistry, Medical University of Vienna, Vienna, Austria

**Keywords:** Cerebellar pilocytic astrocytoma, Cystic pilocytic astrocytoma, Extent of cyst wall resection, Cyst wall enhancement

## Abstract

**Purpose:**

We aimed to present our surgical experience and the impact of a solid or cystic morphology of cerebellar pilocytic astrocytoma (cPA) on surgery and the risk for a re-resection.

**Methods:**

We retrospectively analyzed all children operated at our institution between 2009 and 2023 for cPA. Tumours were categorized into 4 groups: (i) cystic PA without cyst wall enhancement, (ii) cystic PA with cyst wall enhancement, (iii) solid tumour, (iv) and solid tumour with central necrosis.

**Results:**

Forty-two children with a median age at surgery of 7.1 years (range 0.7–14 years; male to female ratio 1.5) were identified. The median follow-up time was 3.1 years (0.6–14 years). Twenty-eight patients (66.6%) presented with cystic PA (20 without and 8 with cyst wall enhancement), 9 patients (21.4%) exhibited a solid tumour with central necrosis and 5 (11.9%) had a solid tumour without central necrosis. Gross total resection could be achieved in 31 patients (73.8%), near total resection in 6 (14.3%), and subtotal resection in 5 (11.9%). Progression occurred in 11 cases with 9 patients having a 2nd resection after a mean time of 3.4 years. The highest risk for a 2nd resection was seen in the group of solid tumours with a necrotic centre (odds ratio = 2.3), progression of enhancing cyst wall remnants was seen in one out of two patients with remnants needing reoperation.

**Conclusion:**

Surgery in cerebellar PA should aim for gross total resection of the solid-enhancing tumour.

## Introduction

Pilocytic astrocytoma (PA) is the most common pediatric glioma [1], accounting for approximately 20% of all paediatric brain tumours under 15 years of age and 30% of all paediatric tumours located in the posterior fossa. [1, 3, 4, 10, 15]. An attempt for gross total resection is the mainstay of treatment reaching excellent survival rates of > 90% after 10 years [5]. The rates of achieving gross total resection vary between 67 and 74.1% in recent reports [1, 6, 12]. In case a residual tumour had been left in place either intentionally in case of a given risk for morbidity or unintentionally, tumour progression may occur necessitating re-operation. This need for a second or third resection has been reported to be between 26% and 29% for a second operation and 9.6% for a third operation [1, 12, 13]. The time interval between first resection and re-operation ranges between 6 and 92 months [12].

Factors that attribute to the risk of incomplete resection, progression, and the need for re-operation or non-surgical treatments have been scarcely addressed in the literature so far. [1] The size of the tumour and its growth pattern have been identified as contributing factors with unilateral hemispheric location and cystic components to be favourable [7, 16]. Rare cases of malignant transformation were anecdotally reported usually after radiation therapy [10].

The need to resect the cyst wall of cerebellar pilocytic astrocytomas (cPA) in order to reduce the risk of recurrence and the need for further surgical or non-surgical interventions have not been systematically investigated. In general, it seems well accepted, however, that non-enhancing cysts of cPA do not warrant resection. For enhancing cyst walls this is less clear. As systematic histological evaluations of cyst walls are missing, the question remains whether these exhibit tumour cells and represent a potential source of tumour progression or recurrence [2]. Therefore, we thought to analyse our patient population specifically addressing the pattern of recurrence and progression in cerebellar pilocytic astrocytoma based on their growth pattern and morphological type.

## Methods

We performed a retrospective study of 42 patients operated between 2009 and 2023 for a cPA at the Department of Neurosurgery of the Medical University of Vienna. We excluded patients with exophytic brainstem tumours.

The following data was extracted from the medical charts: demographic information, clinical presentation, tumour configuration and preoperative tumour volume, extent of resection, postoperative complications, progression/recurrence rate with times to progression/recurrence, metastases, need to perform further surgical or non-surgical treatment, neurological status at the last follow-up.

Time to progression/recurrence was defined as the time between the operation and the first MRI verifying progression/recurrence.

**Fig. 1:**
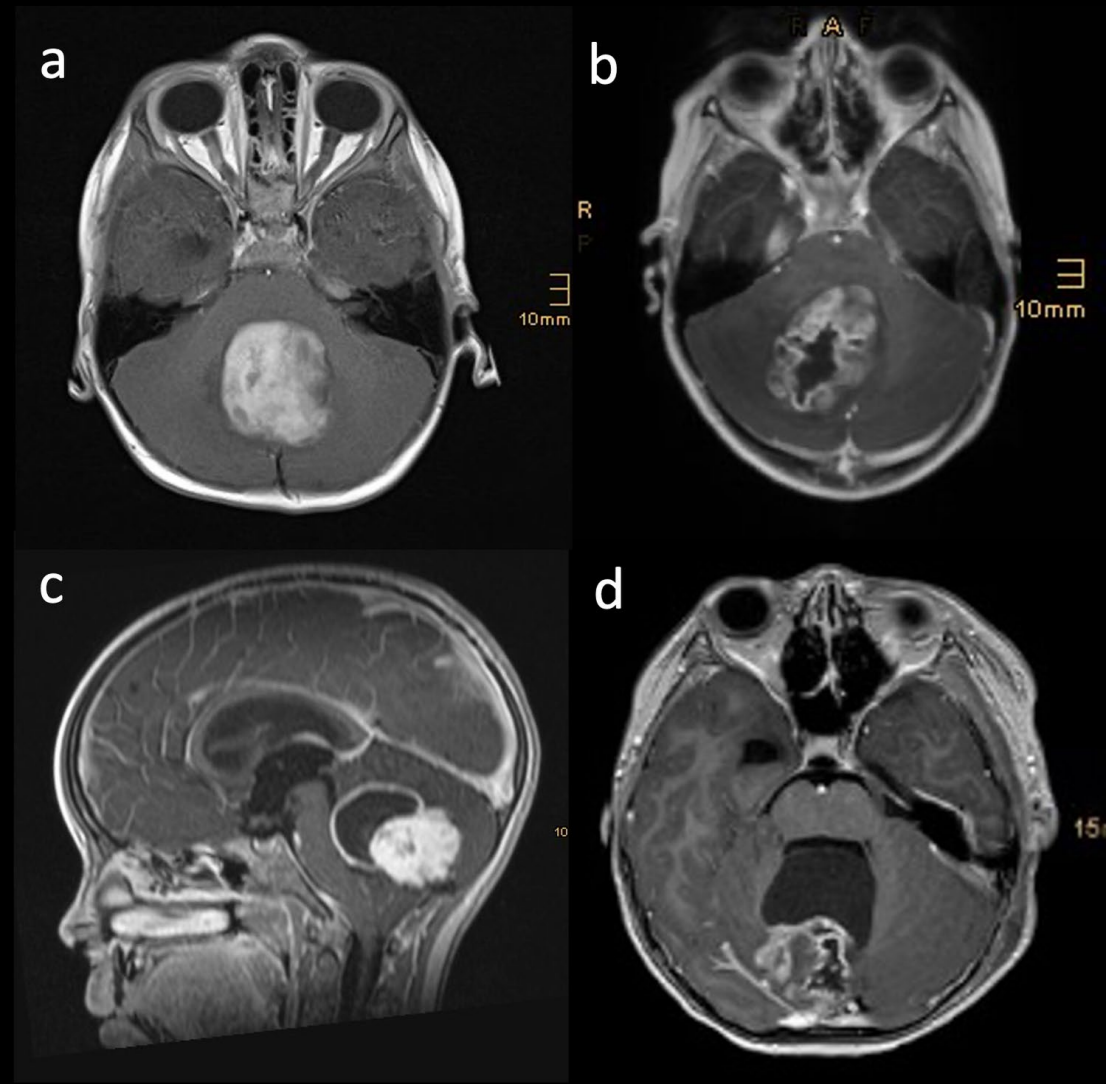
**a** Solid tumour. **b** Solid tumour with central necrosis. **c** Cystic tumour with enhancing cyst wall. **d** Cystic tumour with non-enhancing cyst wall

Patients were categorized into four groups based on the tumour morphology on preoperative MRI as suggested by Zakrzewski et al. [17]: cystic tumour with a non-enhancing cyst wall, cystic tumour with an enhancing cyst wall, solid tumour with central necrosis and solid or mainly solid tumour (Fig. 1).

The extent of resection was categorized into GTR (100%), NTR (> 90%), and STR (< 90%) divided by solid and cystic components. The grade of resection was defined based on pre- and postoperative T1-weighted post-contrast MRI. Non-enhancing cyst walls have never been resected at our institution throughout the study period. In tumours with enhancing cyst walls, it has always been intended to resect the cyst wall. Calculations of the tumour and cyst volumes were performed using IMPAX volumetry tool. Systematic histological evaluations of the resected cyst walls have not been performed.

Follow-up management at our institution consisted of clinical follow-up visits and MRI evaluations at 3 months after surgery and then every 6 months for 5 years.

Neurological status at the last follow-up was used for the analysis. Three patients who developed new neurological deficits during the follow-up time attributed to other comorbidities (other CNS tumours and autism) were excluded from this analysis.

### Statistical analysis

Correlations between the parameters were calculated using Fisher’s exact tests. The level of statistical significance was set at *P* < 0.05. Due to a small sample size, odds ratios were used additionally. The follow-up period was calculated from the date of tumour resection to the date of relapse or regrowth of the residual tumour or the last contact with the patient. Statistical analysis was performed with SPSS and Excel Software.

## Results

In our cohort of 42 patients, the median age at the time of surgery was 7.1 years (range 0.7–14 years) with a male to female ratio of 1.5 (25 males, 17 females). Two patients (4.7%) had NF1. The median follow-up time was 3.1 years (range 0.6 –14 years).

The most common symptoms at presentation were clinical signs of raised intracranial pressure (76%) and ataxia (45%). Other symptoms were diplopia (4), strabismus (3), motor weakness (2), and nystagmus (1).

An external ventricular drain (EVD) was placed in 11 patients before or during the first operation (preoperatively *n* = 7, intraoperatively *n* = 4). The mean time with the EVD in situ was 6.8 days. In one patient, an EVD was placed during the second operation. Subsequent shunt placement was necessary in two children (4.8%).

Of 42 patients, 38 had a PA located exclusively in the cerebellum, while five patients had some 4th ventricular involvement. 16 tumours were located solely in the right hemisphere, 13 in the left, and three in the vermis. In the remaining five patients given an extensive tumour volume the vermis and the hemispheres were affected to a varying extent.

Twenty patients (47.6%) had a cystic PA without cyst wall enhancement, 8 patients (19%) a cystic PA with cyst wall enhancement; 9 patients (21.4%) exhibited a solid tumour with central necrosis and 5 (11.9%) had a solid tumour without central necrosis. The median tumour volume was 57.6 cm^3^, in cystic tumours the median size of the cyst was 43.5 cm^3^ with the median cystic to solid component ratio of 0.6. In 11 children an obstructive hydrocephalus was present.

### Grade of solid and cystic tumour resection, progression and reoperation rates

At first operation, GTR could be achieved in 31 patients (73.8%), NTR in 6 patients (14.3%), and STR in 5 patients (11.9%). In four patients with cystic tumours a solid tumour remnant was detected with a mean volume of 1.9 cm^3^ (range from 0.3 to 3.7 cm^3^). Two additional patients harboured enhancing cystic wall remnants.

We compared the resection rates estimated by the surgeon with the postoperative MRI. In 31 patients, the surgeon’s impression correlated with the postoperative MRI, in 5 cases the surgeon overestimated the resection rate, in 5 patients the surgeon was not sure about the grade of resection and in the remaining case an intraoperative MRI was performed revealing GTR.

**Fig. 2:**
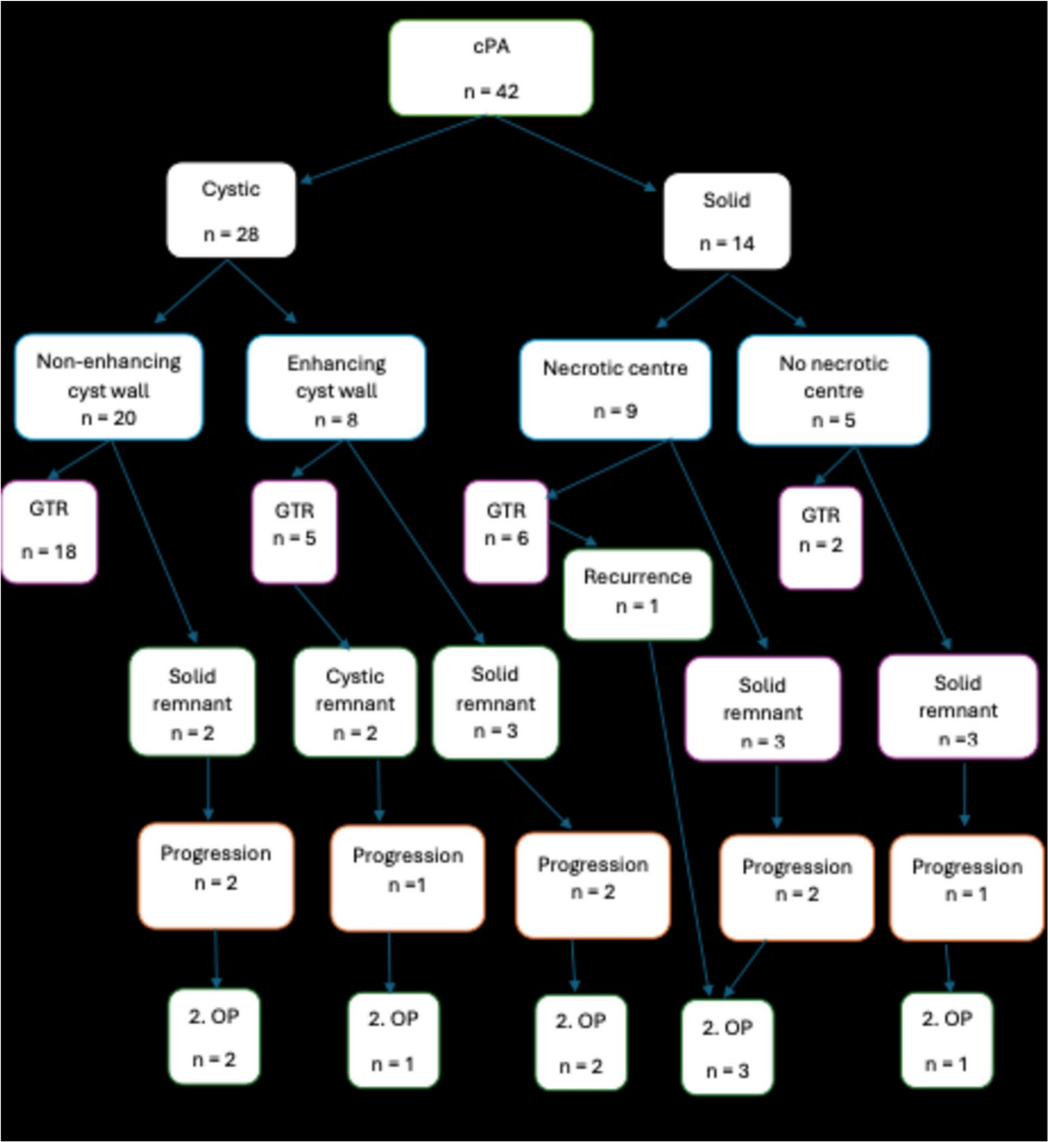
Grade of resection, type of tumour remnant, progression/recurrence and the need to perform a second resection by morphologic tumour type

Progression occurred in 11 cases. Tumour recurrence was observed in one patient after complete resection. Details based on tumour morphology can be found in Table 2. A second resection was performed in 9 patients after a median time of 2.6 years [0.3–9.2 Years]. Of these 9 patients one was a recurrence after a complete resection, 8 were progressions of remnants, of which 5 had progression after STR, 2 after NTR, and 1 of an enhancing cyst wall remnant. Progression of the remnant was symptomatic only in one patient due to aqueductal stenosis, leading to a prompt reoperation. In all other cases the second resection was performed for asymptomatic progression detected on routine follow-up MRI. After 2nd resection, further progression was seen in one patient after 2nd incomplete resection, necessitating a third operation after 4.2 years from 2nd surgery. Data on the grade of resection, type of remnant, progression/recurrence and the need to perform the second operation by morphologic tumour type is summarized in Fig. 2.

In the group of tumours with a non-enhancing cyst wall, there were 2 cases of solid remnants in addition to the 18 cyst wall remnants. While 15 patients with a cyst wall remnant showed no progression over time, 3 patients had some insignificant asymptomatic pseudoprogression of the cyst remnant for which a second surgery was deemed unnecessary. On the contrary, both patients from this group with a solid remnant had a tumour progression over time and needed a subsequent re-resection. One of these patients showed a BRAF fusion.

In the group of cystic tumours with enhancing cyst wall, a different trend could be observed: out of 2 cases with a cystic remnant, one had a progression prompting a second resection. From the three cases with a solid remnant, two showed progression and were re-operated.

The median time to progression after the first surgery was 1.37 years. The longest median time to progression was observed in the group of cystic tumours with non-enhancing cyst walls. Times to recurrence/progression and remnant characteristics by morphologic tumour type are summarized in Table 1. The second surgery was performed in three patients who had a remnant of solid tumours with necrotic centre, one patient with a remnant of a solid tumour and 3 patients with a cystic (*n* = 1) or solid (*n* = 2) remnant of a cystic tumour with enhancing cyst walls.

**Table 1:** Progression and time to recurrence after first resection by radio-morphologic group of cPA

Tumour type	Solid remnant	Cyst wall remnant	Progression/recurrence	Time to progression/recurrence (in years)
Cystic with enhancing wall, *n* = 8	3	2	3 progression	1.1
Solid with necrotic centre, *n* = 9	3	0	2 progression, 1 recurrence	0.7 progression, 0.8 recurrence
Solid, *n* = 5	3	0	1 progression	0.4

Of the four radio-morphologic groups of cPA the highest OR (odds ratio) of the need to perform the second operation was observed in the group of solid tumours with necrotic centre (OR= 2.3) and the lowest in the group of cystic tumours with non-enhancing cyst wall (OR = 0.27). In cystic tumours, the higher rate for the need to perform a second resection was observed in the group of cPA with enhancing cyst wall (OR = 2.25). The effect is even more pronounced when comparing the two groups of cystic tumours only (OR = 4.25). Data on the type of remnants in cystic PA with or without the need for second surgery are shown in Table 2. Considering patients with tumours and enhancing cyst walls only, three patients had a solid remnant for which two needed a 2nd resection and two patients harboured a cystic remnant of which one showed progression and needed a 2nd resection.

**Table 2:** Cystic PA with solid or cystic remnants

Pat. Nr	Type of remnant (in cm^3^)	1 = w/o enhancement, 2 = with enhancement)	2nd resection
1	Solid (0.9)	1	+
6	Solid (2)	2	-
16	Cyst wall	2	-
19	Cyst wall	2	+
28	Solid (0.3)	2	+
33	Solid (3.7)	1	+
34	Solid (1.8)	2	+

Younger age at the time of the surgery had a significant effect on the need for a re-operation (*p* = 0.011, regression coefficient − 0.36).

Tumour volume, cyst volume, and ratio of cystic to solid components had no significant influence on the need to perform a re-resection.

One patient exhibited spinal drop metastases. This patient had a solid tumour with ventricular involvement. In both patients with NF1 a complete resection was achieved with no recurrence during follow-up. Tumour morphology was solid in one case and solid with necrotic centre in the second.

In this cohort, there was no patient who underwent radiotherapy. One patient underwent chemotherapy with vincristine and carboplatin after three operations.

### Surgical complications

One patient experienced a massive bifrontal epidural hematoma from a skull fracture caused by the head clamp, which needed emergent evacuation. He recovered well without any neurological compromise. One patient had a small PICA stroke, which was clinically uneventful. There were no cerebrospinal fluid (CSF) leaks or wound complications encountered in this series.

### Outcome

After a median time of 3.1 years all patients were alive at the last follow-up.

Of the 39 patients included in the outcome analysis at the time of the last follow-up, 15 children (38%) had no neurological deficits, while 24 (62%) exhibited some mild symptoms. Most commonly, these deficits included ataxia, diplopia, and nystagmus. There were no cases of cerebellar mutism in our cohort.

The highest rate of mild neurological disability was observed in the group of cystic tumours with enhancement of the cyst wall. The data on the neurological outcome by tumour morphology is shown in Table 3.

**Table 3:** Neurological outcome by tumour morphology

Tumour morphology	No deficits	Mild deficits
Cystic w/o enhancement, *n* = 19	7 (36.8%)	12 (63.2%)
Cystic with enhancement, *n* = 7	2 (28.6%)	5 (71.4%)
Solid with necrotic centre, *n* = 8	4 (50%)	4 (50%)
Solid, *n* = 5	2 (40%)	3 (60%)

There was no significant correlation between age at the time of operation and neurological deficits.

## Discussion

Our study corroborates with previous findings showing that GTR often leads to a complete remission. At the same time, incomplete resection did not necessarily lead to progression and a need to perform a re-resection as remnants can either regress, do not progress, or progress at a very slow rate [1, 3, 8, 11, 14]. Adding to this, our analysis indicates that inclusion of the enhancing cyst wall into the resection might be beneficial while non-enhancing cysts do not warrant removal.

**Fig. 3:**
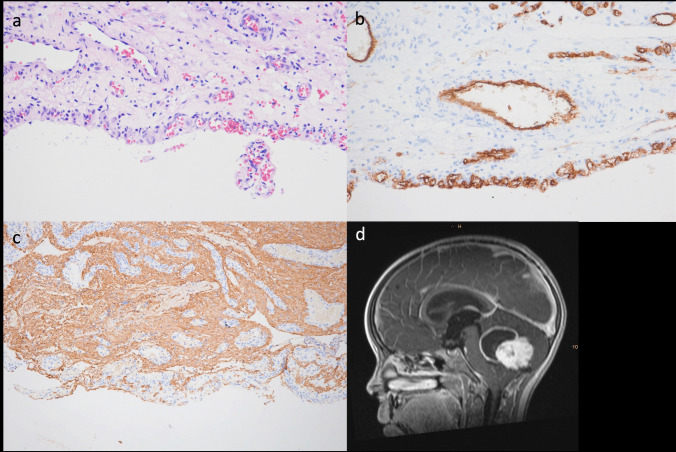
**a** H&E immunohistochemistry staining of the cyst wall. **b** CD34 immunohistochemistry staining showing garland-like vascular proliferation within the cyst wall. **c** GFAP immunohistochemistry staining depicting the glial tumour cells within the cyst wall. **d** Sagittal T1-weighted contrast-enhanced MRI showing the cystic tumour with contrast enhancement of the cyst wall

Previous studies addressing the question of whether cyst wall removal is beneficial or not are very limited including only a very small number of patients. Beni-Adani et al., for instance, looked at three of their patients with two of them located supratentorially, suggesting that enhancing cyst walls can be left in place without increasing the need for a re-resection [2]. In a series by Pencalet et al. contrast enhancement or its absence did not accurately reflect the presence or absence of tumour in the cyst wall [11]. Tumour was found in 1 out of ten of the non-enhanced and in 7 out of ten of the enhanced walls. Enhancement in this series, however, was based on computed tomography or MRI scans. A study by Palma et al. reported that the long-term follow-up did not show any advantage of cyst wall removal [9]. However, they included also non-pilocytic histology and patients who had surgery before the MRI-era. In our experience of 42 children, however, the re-resection rate was significantly higher in tumours with enhancing compared to non-enhancing cysts (OR = 4.25). This has important surgical implications. Non-enhancing cyst walls are typically very thin, transparent, and a clear cleavage plane to the surrounding cerebellum is missing. Any attempt to dissect these walls from the cerebellum too vigorously puts the patient at risk for postoperative oedema, ischemic events and neurological compromise. Therefore, we feel confirmed by our data that a conservative handling of these cysts is justified as we did not see any recurrence at the site of the non-resected walls in 20 cases. Even though a systematic histological analysis of non-enhancing cyst walls in cPA is lacking, it seems likely to us that these do not harbour tumour cells, but can be more considered exclusively paratumoural. In contrast, enhancing cyst walls often peel off the cerebellum quite easily. Even though we saw only one progression in two patients with enhancing cystic remnants, we will continue to attempt a complete removal of the enhancing cyst wall unless a too vigorous manipulation of critical structures such as the floor of the 4th ventricle, superior cerebellar peduncle, or dentate nucleus is necessary. On the other hand, one needs to consider that the larger the cysts the larger the cerebellar surface needing dissection, which may contribute to neurological compromise and may explain the higher rate of mild deficits seen in our series. Unfortunately, we did not systematically evaluate the resected cyst walls histologically but do have a case of an enhancing cyst wall that showed tumour cells upon histology (Fig. 3).

This attempt for a complete resection in tumours with large cysts is sometimes challenging as the collapsing cyst may prevent a good overview of the situs with time, thereby fostering difficult orientation and hidden parts of the tumour can easily be overlooked. Meticulous planning and anticipation of this scenario are critical to avoid unnecessary large tumour remnants and a somewhat uncontrolled chasing of the tumour once the orientation is diminished. A toovigorous manipulation of the cerebellum and the tumour cavity may cause unnecessary injury. At the same time, a controlled and slow drainage of the cyst through a catheter before dural opening allows for a relaxed posterior fossa also avoiding the need for a ventricular drainage through a frontal burr hole in most cases. This ability to have an immediate release of the pressure within the posterior fossa and subsequent tension-free surgery may also contribute to the fact that patients with purely solid tumours experience more neurological compromise in the literature [16]. In our series of 42 children, however, we did not see this difference, which may be explained by the fact, that in large solid tumours causing hydrocephalus, it had been our concept to put in an EVD to guarantee a relaxed posterior fossa at the time of surgery. We strongly believe that having a relaxed posterior fossa at the time of surgery is the mainstay also in surgery for cPA.

With regard to the solid tumour parts, the impact of the morphological type of the tumour on recurrence and relapse was already discussed by previous authors. Similar to our findings, Kameda-Smith et al., for instance, saw that solid tumours are more likely to recur/progress and also exhibit a shorter time to progression/recurrence [8]. In our analysis dividing the tumours into 4 morphological types, the presence of a necrotic part was specifically associated with a higher chance for progression making a re-resection necessary. (OR = 2.3) These different patterns may indicate also differences in the molecular biology. However, in a study by Zakrewski et al., it had been shown that the tumour location, but not the morphology had an influence on the transcriptional profiles of the tumour [17]. Likewise, Kameda-Smith et al. suggested, that cPA with the involvement of the 4th ventricle may be biologically different from the rest of the PA [8]. Our data would support this notion as the reoperation rate was also higher for tumours involving the 4th ventricle compared to other locations. At the same time, this may be explained simply by the fact that a higher rate of STR and residual tumour volume occur when operating at/near this critical area. Furthermore, our follow-up times vary largely limiting definite conclusions.

Future collaborative efforts are needed to further characterize cPA morphological types with regard to potential differences in their molecular signatures in order to advance the field and maybe individualize the surgical strategy for cPA. Especially the influence of the BRAF status on morphology and risk for recurrence needs to be further scrutinized.

## Conclusion

Surgery for cerebellar PA should aim for gross total resection of the enhancing solid tumour. The definite benefit of including the enhancing cyst walls into the resection needs further assessment. Non-enhancing cyst walls do not warrant resection.

## Data Availability

No datasets were generated or analysed during the current study.

## References

[1] Becker AP, Santos de Oliveira R, Saggioro FP, Neder L, Chimelli LMC, Machado HR (2010) In pursuit of prognostic factors in children with pilocytic astrocytomas. Child’s Nervous System 26(1):19–28. 10.1007/s00381-009-0990-8 10.1007/s00381-009-0990-8 19823847

[2] Beni-Adani L, Gomori M, Spektor S, Constantini S (2000) Cyst wall enhancement in pilocytic astrocytoma: neoplastic or reactive phenomena. Pediatr Neurosurg 32(5):234–9. 10.1159/000028944 10965269 10.1159/000028944

[3] Ceppa EP, Bouffet E, Griebel R, Robinson C, Tihan T (2007) The pilomyxoid astrocytoma and its relationship to pilocytic astrocytoma: report of a case and a critical review of the entity. J Neurooncol 81(2):191–6. 10.1007/s11060-006-9216-z 16850101 10.1007/s11060-006-9216-z

[4] Colin C, Padovani L, Chappe C, Mercurio S, Scavarda D, Loundou A, Frassineti F, Andre N, Bouvier C, Korshunov A, Lena G, Figarella-Branger D (2013) Outcome analysis of childhood pilocytic astrocytomas: a retrospective study of 148 cases at a single institution. Neuropathol Appl Neurobiol 39(6):693–705. 10.1111/nan.12013 23278243 10.1111/nan.12013

[5] Collins VP, Jones DT, Giannini C (2015) Pilocytic astrocytoma: pathology, molecular mechanisms and markers. Acta Neuropathol 129(6):775–88. 10.1007/s00401-015-1410-7 25792358 10.1007/s00401-015-1410-7 PMC4436848

[6] Elwatidy SM, Ahmed J, Bawazir MH, Alnasser A, Abanumy J, Al Shammari A, Alduhaish A, Malik SH, Elwatidy HS (2022) Outcome of childhood cerebellar pilocytic astrocytoma: a series with 20 years of follow up. Cureus 14(2):e22258. 10.7759/cureus.22258 35350495 10.7759/cureus.22258 PMC8933261

[7] Fernandez, Figarella-Branger D, Girard N, Bouvier-Labit C, Gouvernet J, Paz Paredes A, Lena G. (2003) Pilocytic astrocytomas in children: prognostic factors--a retrospective study of 80 cases. Neurosurgery 53(3):544-5310.1227/01.neu.0000079330.01541.6e 10.1227/01.neu.0000079330.01541.6e 12943571

[8] Kameda-Smith MM, Green K, Hutton DL, Jeelani NUO, Thomppson D, Hargrave D, Aquilina K (2024) The role of reoperation in pediatric cerebellar pilocytic astrocytoma. J Neurosurg Pediatr 34(2):169–175. 10.3171/2024.2.Peds23236 38759245 10.3171/2024.2.PEDS23236

[9] Palma L, Celli P, Mariottini A (2004) Long-term follow-up of childhood cerebellar astrocytomas after incomplete resection with particular reference to arrested growth or spontaneous tumour regression. Acta Neurochir (Wien) 146(6):581–8. 10.1007/s00701-004-0257-9 15168226 10.1007/s00701-004-0257-9

[10] Parsa CF, Givrad S (2008) Juvenile pilocytic astrocytomas do not undergo spontaneous malignant transformation: grounds for designation as hamartomas. Br J Ophthalmol 92(1):40–6. 10.1136/bjo.2007.125567 17962395 10.1136/bjo.2007.125567

[11] Pencalet P, Maixner W, Sainte-Rose C, Lellouch-Tubiana A, Cinalli G, Zerah M, Pierre-Kahn A, Hoppe-Hirsch E, Bourgeois M, Renier D (1999) Benign cerebellar astrocytomas in children. J Neurosurg 90(2):265–73. 10.3171/jns.1999.90.2.0265 9950497 10.3171/jns.1999.90.2.0265

[12] Ruella M, Giovannini S, Pirozzi Chiusa C, Perez Zabala J, Argañaraz R, Mantese B (2023) Cerebellar pilocytic astrocytoma. Retrospective cohort study assessing postoperative functional outcome, cerebellar mutism and hydrocephalus. World Neurosurg X 19:100180. 10.1016/j.wnsx.2023.100180 10.1016/j.wnsx.2023.100180 PMC10068610 37021292

[13] Stüer C, Vilz B, Majores M, Becker A, Schramm J, Simon MC (2007) Frequent recurrence and progression in pilocytic astrocytoma in adults. Cancer 110(12):2799–808. 10.1002/cncr.23148 17973253 10.1002/cncr.23148

[14] Sutton LN, Cnaan A, Klatt L, Zhao H, Zimmerman R, Needle M, Molloy P, Phillips P (1996) Postoperative surveillance imaging in children with cerebellar astrocytomas. J Neurosurg 84(5):721–5. 10.3171/jns.1996.84.5.0721 8622142 10.3171/jns.1996.84.5.0721

[15] Tabash MA (2019) Characteristics, survival and incidence rates and trends of pilocytic astrocytoma in children in the United States; SEER-based analysis. J Neurol Sci 400:148–152. 10.1016/j.jns.2019.03.028 30953904 10.1016/j.jns.2019.03.028

[16] Villarejo F, de Diego JM, de la Riva AG (2008) Prognosis of cerebellar astrocytomas in children. Childs Nerv Syst 24(2):203–10. 10.1007/s00381-007-0449-8 17710415 10.1007/s00381-007-0449-8

[17] Zakrzewski K, Jarzab M, Pfeifer A, Oczko-Wojciechowska M, Jarzab B, Liberski PP, Zakrzewska, (2015) Transcriptional profiles of pilocytic astrocytoma are related to their three different locations, but not to radiological tumor features. BMC Cancer 15(1):778. 10.1186/s12885-015-1810-z 26497896 10.1186/s12885-015-1810-z PMC4619381

